# Psoas haematoma as a complication of Veress needle insertion: description of a case and literature review

**DOI:** 10.1186/1471-2482-14-104

**Published:** 2014-12-09

**Authors:** Diana García-Alcázar, Beatriz García-Chapinal, Emma Batllori-Badia, Gregorio López-González, Estela Lorenzo-Hernando, Jesús S Jiménez-López, Leticia Muñoz-Hernando, José Luis Muñoz-González

**Affiliations:** Gynecological Endoscopy Unit, Obstetrics and Gyneacology Service, University Hospital 12 de Octubre, Avda Cordoba s/n 28041, Madrid, Spain

**Keywords:** Veress needle, Psoas, Haematoma, Accidental vascular injury

## Abstract

**Background:**

In terms of gynaecological laparoscopic surgery, major complications affecting great vessels, and especially the retroperitoneal ones, are unusual.

**Case presentation:**

We introduce a case of a retroperitoneal haematoma associated with psoas muscle pseudoaneurysm, as a side effect of Veress needle insertion, during laparoscopic surgery. Such complication was managed conservatively at first, requiring finally arterial embolisation.

**Conclusion:**

Even though potential complications associated with laparoscopic surgery are infrequent, they must not be underestimated, and in some cases might need a multidisciplinary management.

## Background

Laparoscopy has turned into the usual procedure used for diagnostic as well as for therapeutic purposes. As a minimally invasive technique, it has become the chosen method for most abdominal processes requiring surgery. Nevertheless, it is not risk-free. In this clinical case, we describe a case of psoas muscle haematoma associated with a pseudoaneurysm of the fourth lumbar artery as an exceptional complication after Veress needle puncture. This is the first case reported in our hospital. We have not found any similar references in literature.

## Case presentation

We report a case of a 32-year-old woman, smoker, with a BMI 20 (weight:55 kg, height: 166 cm) and a personal history of 4 years of primary sterility due to endometriosis with failure after one IVF cycle, and surgical history of two uncomplicated laparoscopic ovarian cystectomies and appendectomy. A 4 cm endometrioma on the left ovary was recently diagnosed; therefore a new laparoscopic procedure was suggested to improve the success rate of a new IVF cycle and also to improve patient’s symptoms such as dyspareunia, dysmenorrea and hypermenorrhea. The pneumoperitoneum was carefully created at the first attempt through Veress needle insertion at Palmer’s point without experiencing any difficulties. When introducing the camera, moderate hemperitoneum was noticed on the left flank and pelvis, without a clear origin.

After aspiration of haematic content and irrigation with physiological saline, bleeding ceased spontaneously, assuming then that the origin was possibly a lesion of an omental vessel. The patient maintained haemodynamic stability throughout the whole process. Surgery continued: left ovarian cystectomy was performed and endometriosic nodule was removed at the level of the left uterosacral ligament.

After surgery, patient was transferred to gynaecological postoperative ward in good general health status.

Thirty-six hours post-surgery, while the patient was performing a flexion movement of the lower limbs, an acute and intense feeling of pain began, associated with the extension movement of her left lower limb radiating to lumbar area. After neurological examination, neurological pathology was ruled out and symptoms were related to mechanical muscle pain due to lithotomy posture used during surgery.

**Figure 1:**
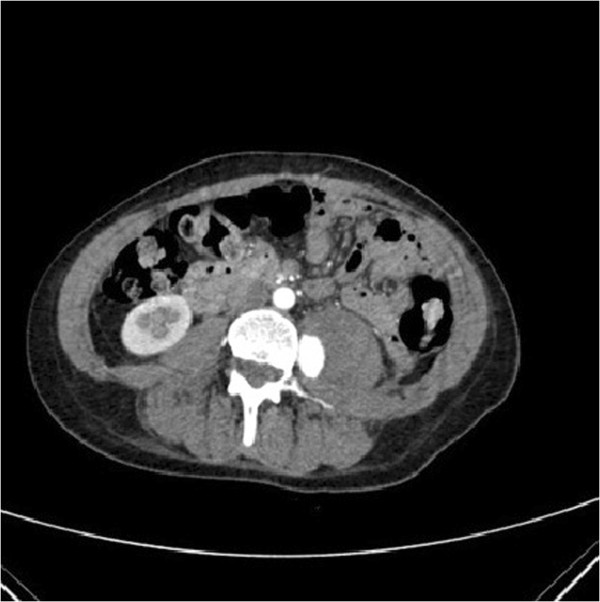
**CT scan image during the excretory phase, which show a left psoas iliac muscle haematoma with an image consistent with a pseudoaneurysm.**

Due to the lack of good pain control in the first 48 hours, an abdominopelvic computed tomography (CT) scan was performed, describing a psoas left muscle haematoma with an image consistent with a pseudoaneurysm of 2,3 × 2,5 × 1,5 cm, on its medial border, at the anatomic level of L4, most probably depending on the lumbar artery (Figure 1). The study was completed by an electromyography (EMG), being diagnosed with left femoral and left femoral cutaneous nerve compression injury.

**Figure 2:**
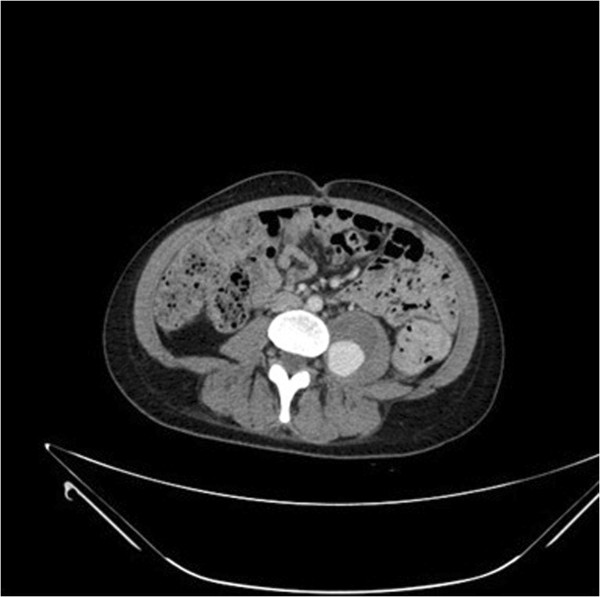
**CT Angiogram with signs of embolisation of the pseudoaneurysm of the left lumbar artery L4, with filling of the aneurismal sac.**

**Figure 3:**
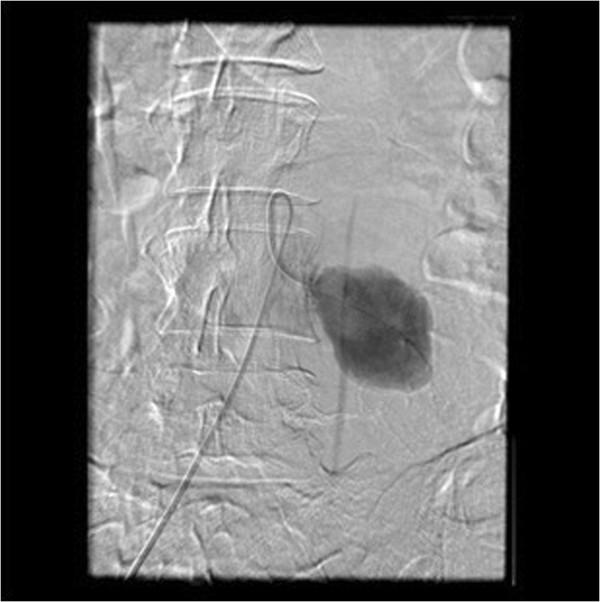
**CT Angiogram: metallic device from the embolisation of the pseudoaneurysm of the left lumbar artery.**

Initially, an expectant management was agreed among the Vascular Surgery, Interventional Radiology and Neurology services aimed at controlling clinical symptoms by means of medical treatment and physical therapy, and by imaging-monitoring the progress of the haematoma. After one month of conservative management without any significant or functional clinical improvement, the patient was reassessed by a new CT scan, which showed a slight increase in the size of the pseudoaneurysm (2,5 × 2,5 cm). After reassessing the case together with the Vascular Surgery and Interventional Radiology services, an arteriography of the left lumbar artery and its subsequent embolisation were performed. A CT Angiogram after 30 days proved vascular leakage, therefore a second successful embolisation was done (Figures 2 and 3).

During the follow-up, the patient needed various drugs in order to achieve pain control, including opioids. Six months after the onset of the clinical figure, the need for analgesia had progressively decreased, and one year after, only certain discomfort persists with no need for analgesic medication; therefore the patient has been discharged from Pain Control Clinic. From the neurological point of view, and after the completion of the physical therapy, a significant but incomplete improvement in strength is noticed (on physical examination the strength in psoas, quadriceps and adductor muscles is 4/5), the patient is able to walk between 30 and 60 minutes without feeling any pain, and passive movements are preserved but no ability to actively elevate lower limb during extension above 20° is observed. Therefore, the stage of sequelae has not yet been overcome. In terms of gynaecological recovery, both clinical and ultrasound examination became normal and the patient wishes to initiate a new IVF cycle.

**Figure 4:**
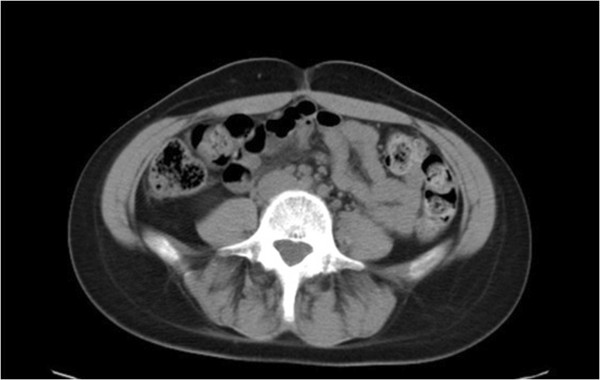
**Abdominopelvic CT scan where the complete resolution of the haematoma can be observed.**

The EMG shows a significant recovery of the nerve injury and CT scan demonstrates a complete resolution of the haematoma (Figure 4).

## Discussion

Since Veress needle invention in 1938, its usage has been gaining in popularity and nowadays, it has been spread worldwide. The creation of pneumoperitoneum is the moment of maximum risk of complications in laparoscopic surgery, mainly due to abdominal viscera and great abdominal vessels lesions [1].

In literature, the majority of vascular complications are due to lesions of great vessels (vena cava, aorta) and they are mostly described during general surgery procedures (appendectomy, colecistectomy) [2–4], and urology procedures with retroperitoneal access [5].

Additionally, gynaecological complications described during gynaecological surgery are normally related to great pelvic vessels (final portions of aorta, cava and iliac main vessels) [6]. In these cases there are high rates of laparotomy reconversion for the correct management of the lesion.

In relation to surgeons’ experience, there were no complications so far such as the one described in the case. In our service, with 25 years of experience in laparoscopic surgery, the following complications have been described in relation to Veress needle insertion: a renal puncture in a monorrenal patient with a hypertrophic pelvic kidney, a gastric puncture in a patient with badly placed nasogastric tube, and a case of iliac vessels puncture with Veress needle inserted at the umbilical point. In the last case, it was necessary to reconvert urgently to laparotomy for the complete resolution of the injury.

There are three techniques for the creation of pneumoperitoneum: closed technique with Veress needle, open technique with Hasson trocar and direct trocar insertion. Even though the chosen method may depend on the surgeon experience, some studies have shown that the most used technique is Veress needle insertion [7, 8]. It is introduced blindly in the abdomen, followed by a safety test which confirms the pneumoperitoneum. After suitable gas insuflation, the main trocar is introduced and subsequently Veress needle is withdrawn under direct vision. The prevalence of lesions described in literature is 0.23% [9]. The most serious complication, which accounts for most of the mortality, is the lesion of great vessels (aorta, vena cava and iliac vessels) [10]; which represents approximately 2.6% of the total lesions.

As regards the insertion site of Veress needle, the entrance can be achieved supraumbilically or at Palmer’s point; based on an extensive bibliographic search, we concluded that the majority of studies which compare different entrance methods use the supraumbilical point to insert Veress needle [11–13]. Palmer’s point is located 3 cm below the left costal margin in the mid-clavicular line [14]; it is essential to decompress the stomach using nasograstric tube suction and insert Veress needle perpendicular to the skin. Lifting the abdominal wall with towel clips during needle insertion can also help to ensure a safe access. This technique is especially recommended in patients with previous surgeries (suspected peritoneal adhesions), obese or very thin patients (short distance from abdominal wall to retroperitoneal vessels), because it seems to reduce the risk of lesions [15].

Psoas muscle puncture with Veress needle is an exceptional complication during surgery, therefore it is easily unnoticed. This type of haematoma is normally related to trauma [16, 17], but in some cases may appear as a spontaneous haematoma; mainly if patients are under anticoagulation therapy [18], haemophilic patients or in young patients affected by arteriovenous malformations [19]. In our case, haematoma was produced by accidental puncture of the left lumbar artery with Veress needle; going unnoticed during surgery and being diagnosed 48 hours after the intervention, by means of imaging techniques.

In our patient, there was a sudden onset of clinical symptoms, causing motor and sensitive dysfunction. Pain started in lumbar area radiating to the lower limb, according to the distribution of the femoral nerve. Although in our case the definite diagnosis was made by CT scan, the gold standard in these cases is the MRI scan [20], which is more sensitive to detect minor haematomas.

Treatment decision about this clinical entity depends on the speed of the onset, the size of the haematoma and the degree of neurological impairment. In cases of small haematomas and moderate neurological symptoms, conservative management consisting of bed rest and analgesia is advised [16]; whereas bigger haematomas with severe neurological impairment require surgery for decompression and drainage [18]. Positive outcome is normally achieved if the treatment is customized. Tamai et al. in a review of published cases, concluded that the prognosis for femoral nerve recovery after haematoma compression was very good [21].

The period of time for complete recovery ranges between months and years, although the average time is 6 months. The patient presented in our case was initially managed conservatively, and only after a month of follow-up without clinical and imaging improvement, active treatment by arterial embolisation was provided.

## Conclusion

Even though potential complications associated with laparoscopic surgery are infrequent, they must not be underestimated. We have to think of the complications according to clinical symptoms and in some cases they may require a multidisciplinary approach.

## Consent

Written informed consent was obtained from the patient for publication of this Case report and any accompanying images. A copy of the written consent is available for review by the Editor of this journal.
